# Hit‐and‐run trophallaxis of small hive beetles

**DOI:** 10.1002/ece3.1806

**Published:** 2015-11-06

**Authors:** Peter Neumann, Jan Naef, Karl Crailsheim, Robin M. Crewe, Christian W. W. Pirk

**Affiliations:** ^1^ Social Insect research GroupDepartment of Zoology and EntomologyUniversity of Pretoria0002PretoriaSouth Africa; ^2^ Institute of Bee HealthVetsuisse FacultyUniversity of Bern3003BernSwitzerland; ^3^ Institut für ZoologieUniversität GrazUniversitätsplatz 28010GrazAustria

**Keywords:** *Aethina tumida*, *Apis mellifera*, honey bee, host–parasite interaction, small hive beetle, solicitation, trophallaxis

## Abstract

Some parasites of social insects are able to exploit the exchange of food between nestmates via trophallaxis, because they are chemically disguised as nestmates. However, a few parasites succeed in trophallactic solicitation although they are attacked by workers. The underlying mechanisms are not well understood. The small hive beetle (=SHB), *Aethina tumida*, is such a parasite of honey bee, *Apis mellifera*, colonies and is able to induce trophallaxis. Here, we investigate whether SHB trophallactic solicitation is innate and affected by sex and experience. We quantified characteristics of the trophallactic solicitation in SHBs from laboratory‐reared individuals that were either bee‐naïve or had 5 days experience. The data clearly show that SHB trophallactic solicitation is innate and further suggest that it can be influenced by both experience and sex. Inexperienced SHB males begged more often than any of the other groups had longer breaks than their experienced counterparts and a longer soliciting duration than both experienced SHB males and females, suggesting that they start rather slowly and gain more from experience. Successful experienced females and males were not significantly different from each other in relation to successful trophallactic interactions, but had a significantly shorter soliciting duration compared to all other groups, except successful inexperienced females. Trophallactic solicitation success, feeding duration and begging duration were not significantly affected by either SHB sex or experience, supporting the notion that these behaviors are important for survival in host colonies. Overall, success seems to be governed by quality rather than quantity of interactions, thereby probably limiting both SHB energy investment and chance of injury (<1%). Trophallactic solicitation by SHBs is a singular example for an alternative strategy to exploit insect societies without requiring chemical disguise. Hit‐and‐run trophallaxis is an attractive test system to get an insight into trophallaxis in the social insects.

## Introduction

Trophallaxis is an integral part of many insect societies and serves to distribute food and integrate information throughout the superorganism (Wilson 1971). Parasites of social insects have repeatedly evolved mechanisms to exploit these trophallactic systems (Schmid‐Hempel 1998). Such parasites have been reported from ants (Hölldobler and Wilson 1990), termites (Howard et al. 1980), and social bees (Ellis et al. 2002a). Most parasites exploiting trophallaxis in social insects rely almost exclusively on chemical mimicry to avoid aggression by host workers and may use acoustical mimicry to elevate their status toward the highest attainable position within their host's social hierarchy and finally use tactile stimuli to induce the act of feeding itself (Howard et al. 1980; Moritz et al. 1991; Schmid‐Hempel 1998; D'Ettorre et al. 2002; Barbero et al. 2009). Reports of nonchemically mediated trophallactic solicitation are currently restricted to the cricket *Myrmecophila manni*, which infests western thatching ants (*Formica obscuripes*, Henderson and Akre 1986) and the small hive beetle (=SHB, *Aethina tumida* Murray, Coleoptera: Nitidulidae), which infests honey bee colonies (*Apis mellifera;* Ellis et al. 2002a). Having invaded the host society, these parasites are not adopted nor tolerated. Instead, both parasites are recognized as non‐nestmates and readily attacked by host workers (Henderson and Akre 1986; Elzen et al. 2001; Neumann et al. 2001b). However, they nonetheless succeed in triggering feeding (Henderson and Akre 1986; Ellis et al. 2002a). Therefore, it appears that chemical stimuli are not crucial for triggering trophallactic feeding in hosts, but that tactile stimuli suffice to exploit trophallactic systems of the host.

The SHB was originally described as a parasite and scavenger of honey bee colonies, *Apis mellifera* by Lundie (1940), and the vast majority of studies since then seem to suggest that this species appears to be the primary host (cf. Neumann and Elzen 2004; Neumann and Ellis, 2008). However, a growing number of reports indicate that SHBs are able to exploit a variety of different social bee species; *Austroplebeia australis*: Halcroft et al. 2008, 2011; *Bombus impatiens*: Spiewok and Neumann 2006b; Hoffmann et al. 2008; *Dactylurina staudingeri*: Mutsaers, 2006; *Melipona beecheii*: Peña et al. 2014; *Tetragonula carbonaria*: Greco et al. 2010; Wade 2012; overviews by Neumann and Elzen 2004; Neumann 2015. It is native to sub‐Saharan Africa (Lundie 1940; Schmolke 1974; Neumann and Elzen 2004), where it is usually considered to be a minor pest only (Pirk et al. 2014; Pirk and Yusuf 2015). The SHB was introduced into the USA (1996), Egypt (2000), Australia (2001) and into Europe twice (2004 and 2014, see Neumann and Ellis 2008 for an overview and Mutinelli et al. 2014 for the Italian case). In the United States and Australia, SHBs are now well established as an invasive species and can be considered an economically significant honey bee pest under suitable environmental conditions (Neumann and Elzen 2004; Spiewok et al. 2007).

While a number of different beetles are associated with honey bee colonies (e.g., Nitidulidae: *Cychramus luteus* (Neumann and Ritter 2004); *Glischrochilus fasciatus*,* Lobiopa insularis*,* Epuraea corticina* (Ellis et al. 2008); Cryptophagidae: *Cryptophagus hexagonalis* [Haddad et al. 2008]), the SHB is the only known species to mimic honey bee trophallaxis (Ellis et al. 2002a). Besides that, SHBs feed on honey and pollen stores, bee brood, dead bees and conspecifics (Neumann and Elzen 2004; Spiewok and Neumann 2006a) and can also reproduce on rotten fruit and other alternative food (Ellis et al. 2002b; Buchholz et al. 2008). Why did they shift from easily accessible fruits to less accessible resources in a honey bee colony? There might be benefits for this resource shift, despite the defensiveness of the bees. Honey bee colonies are perennial and therefore a more spatially and temporally reliable resource. Moreover, owing to the defensiveness of the worker bees, the numbers of potential competitors are probably reduced. Finally, absconding (nonreproductive swarming) of colonies is a common feature in populations of African honey bees (Hepburn et al. 1999), resulting in the abandonment of food stores and brood (Spiewok et al. 2006). This implies that a significant quantity of food could become available for the SHBs when these events occur. Indeed, SHBs can induce absconding of host colonies (Ellis et al. 2003a), and SHB reproductive success on protein‐rich pollen and honey bee brood is orders of magnitude higher than that on fruits (Ellis et al. 2002b; Buchholz et al. 2008). However, honey bees recognize SHBs and exhibit defensive behavior against them (Elzen et al. 2001), often driving them to confined places, where they are constantly guarded by bees (Neumann et al. 2001b; Ellis et al. 2003b; Ellis 2005). In these confinements, the SHBs often have no access to food, except conspecifics, but are instead in constant contact with the host bees. Therefore, trophallactic solicitation by the SHB is likely to be an adaptation to confinement by honey bees. Evolving such sophisticated behavioral mimicry, probably via tactile stimuli, enables SHBs to wait inside a host colony for the right opportunity to exploit the rewarding resources of a weak or abandoned host colony. Given that trophallactic solicitation by the SHB is important for survival within host colonies, this behavior should be innate and even bee‐naïve beetles should exhibit the behavior. These latter two points have not been investigated previously.

In any case, it appears that SHB trophallaxis is not fail‐safe (Ellis et al. 2002a). Due to their hard exoskeleton and their turtle‐defense posture (Neumann et al. 2001b), the beetles probably face a rather small risk of injury by the bees that has not yet been quantified. Nevertheless, SHBs pay an energetic cost during the repeated fast advances and retreats that characterize their trophallactic solicitation (Ellis et al. 2002a). We therefore propose that there is selection for increased efficiency of the soliciting interaction, specifically to reduce the number of retreats and their duration. It would be adaptive for SHBs to improve their begging behavior by developing more efficient soliciting strategies. Moreover, female SHBs have higher protein requirements than conspecific males owing to egg production and larger body size (Lundie 1940; Ellis et al. 2002c). During trophallaxis, honey bee workers can transfer either nectar (the content of the honey stomach, very little protein), or jelly (a protein‐rich glandular secretion, Crailsheim 1998), which can be used as an alternative to pollen for ovary activation in honey bees (Schäfer et al. 2006). If SHBs receive jelly from the workers, we would expect females to accept higher risks than males during trophallactic solicitation, reflecting the relatively higher benefit they may get from trophallactic feeding of jelly. In any case, female SHBs tend to be larger compared to males (Ellis et al. 2002c), hence probably requiring more energy.

Here, we studied SHB trophallactic solicitation using an experimental setup that is likely to reflect natural conditions. Specifically, we give a detailed qualitative and quantitative description of the behavior, quantify the SHB risk of injury and test the following hypotheses: (1) trophallactic solicitation of SHBs is innate and does not require any previous exposure to the host; (2) both sexes of the SHB are able to induce trophallactic feeding; (3) experienced SHBs are more efficient at triggering the feeding behavior of honey bee workers than inexperienced ones; and (4) female SHBs are more efficient at triggering the feeding behavior of honey bee workers compared to male SHBs due to their increased need for food.

## Materials and Methods

### Experimental setup

**Figure 1:**
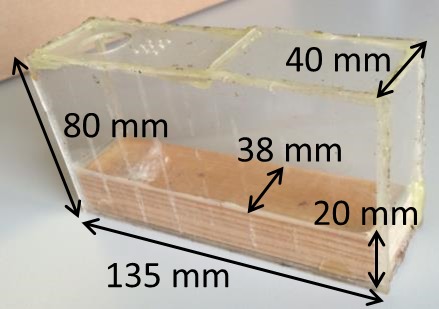
Perspex cages for the experiments. The lower 20 mm of which was restricted to a 2 mm gap by a piece of wood. This gap was wide enough for SHB to enter (Schmolke 1974), but too narrow for the honey bee workers.

Experiments were conducted at the University of Pretoria, South Africa, from June to September 2008. Adult SHBs were collected from local queenright colonies of *A. m. scutellata* and used to initiate a laboratory population reared on honey and pollen following standard protocols (Neumann et al. 2001a; Mürrle and Neumann 2004; Neumann et al. 2013). Emerging adult SHBs were stored in plastic containers supplied with cotton balls soaked in honey and water. Sexually mature individuals (30 days old) were sexed (Schmolke 1974; Neumann et al. 2013) and used in the experiments. Perspex (=clear poly [methyl methacrylate] sheets) cages were used (Fig. 1). Throughout the experiment, the cages were kept in darkness at 34°C and 60% RH and equipped with a feeder providing sugar‐water ad lib to both SHBs and workers (Williams et al. 2013). Four cages were stocked with 10 bee‐naïve SHB females and four with 10 bee‐naïve SHB males. The beetles stayed in the cages for five consecutive days. On each day, 30 newly collected adult honey bee workers from the brood nests of four local *A. m. scutellata* colonies were added to each cage and allowed to settle for 4 h prior to the observations. Then, the cages were filmed for one hour using a Sony HDR‐SR7E camcorder and the workers subsequently re‐collected and released.

### Observations

The recorded AVCHD videos were frame‐served using the free software DGAVCDec 1.0.4 and AviSynth 2.5 and edited with the freeware VirtualDub 1.8.6. Specifically, the videos were cropped to the area of interest, reduced to black and white and brightness and contrast adjusted. The edited videos were encoded with the free Xvid codec 1.1.3 using settings for maximal quality. Behavioral interactions were analyzed from the converted videos using the freeware Elan 3.6.0. Then, the SHB behavior was categorized following Neumann et al. 2013's synthesis of numerous studies (Elzen et al. 2001; Neumann et al. 2001b; Ellis et al. 2002c; Ellis et al. 2004a,b; Ellis 2005; Pirk and Neumann 2013): *Antennating* with a guarding bee (Rösch 1925; Neumann et al. 2001b; Ellis 2005); *Begging*: the SHB approaches the bee's head and drums on the bee's mouth parts with its antennae. Bee moves head and antennae, touching the beetle's pronotum and elytra (Ellis et al. 2002a); *Ignoring*: none of the other behaviors; *Interfering*: obtaining food while taking advantage of another SHB's trophallactic contact; *Retreating*: the SHB experiences aggression from a honey bee or shoving by another SHB and moves away; *Shoving*: the SHB pushes away another SHB with its head; *Trophallactic contact*: obtaining a drop of food from a donor bee. SHB stands still with its mouth parts touching the bee's mouth parts. Bee keeps its head still and touches the SHB's thorax and elytra with its antennae, between 3 and 4 sec; *Turtle‐defense posture*: the SHB stays motionless and tucks its head underneath the pronotum with the legs and antennae pressed tightly to the body; *Success*: We here define SHB success as any sequence of behavioral events resulting in trophallactic feeding by a donor bee.

The first 10 series of the above behaviors separated by at least 5 sec of ignoring were scored for each cage on days 1 and 5. The recorded behaviors were processed with the free spreadsheet software OpenOffice.org Calc 2.3.0. Successive ignoring and turtle‐defense postures were joined and defined as breaks. Series of behaviors, which started with begging and ended with trophallactic contact (=successful) or with begging, followed by a break of at least 5 sec (=unsuccessful), were defined as interactions. Interactions were excluded when (1) interactions from cages, where a bee had squeezed itself into the gap and been fed on by the SHBs (see Pirk and Neumann 2013); and (2) cases for which behaviors could not be scored unambiguously due to poor recording.

### Data analyses

For each interaction, we determined the duration of the trophallactic contact behavior (=feeding duration), the number and mean duration of begging (=begging events and begging duration, respectively), the mean duration of breaks (=break duration), the total of begging and breaks (=soliciting duration), and trophallactic success (whether the interaction results in a trophallactic contact or not). Interactions were only analyzed, when one SHB was involved to reflect individual behavior. As the data did not pass assumptions for normality (Kolmogorov–Smirnoff test), we performed nonparametric Kruskal–Wallis ANOVAs with multiple comparison tests of mean ranks for all groups. Sex (female or male) and experience (inexperienced or experienced) were used as factors. Feeding duration, begging events and begging duration, break duration, soliciting duration, and success were used as dependent variables. In a separate analysis, begging events and begging duration, break duration, and soliciting duration were compared between unsuccessful and successful interactions using Mann–Whitney U‐tests. All analyses were performed using STATISTICA© 12.0.

## Results

**Figure 2:**
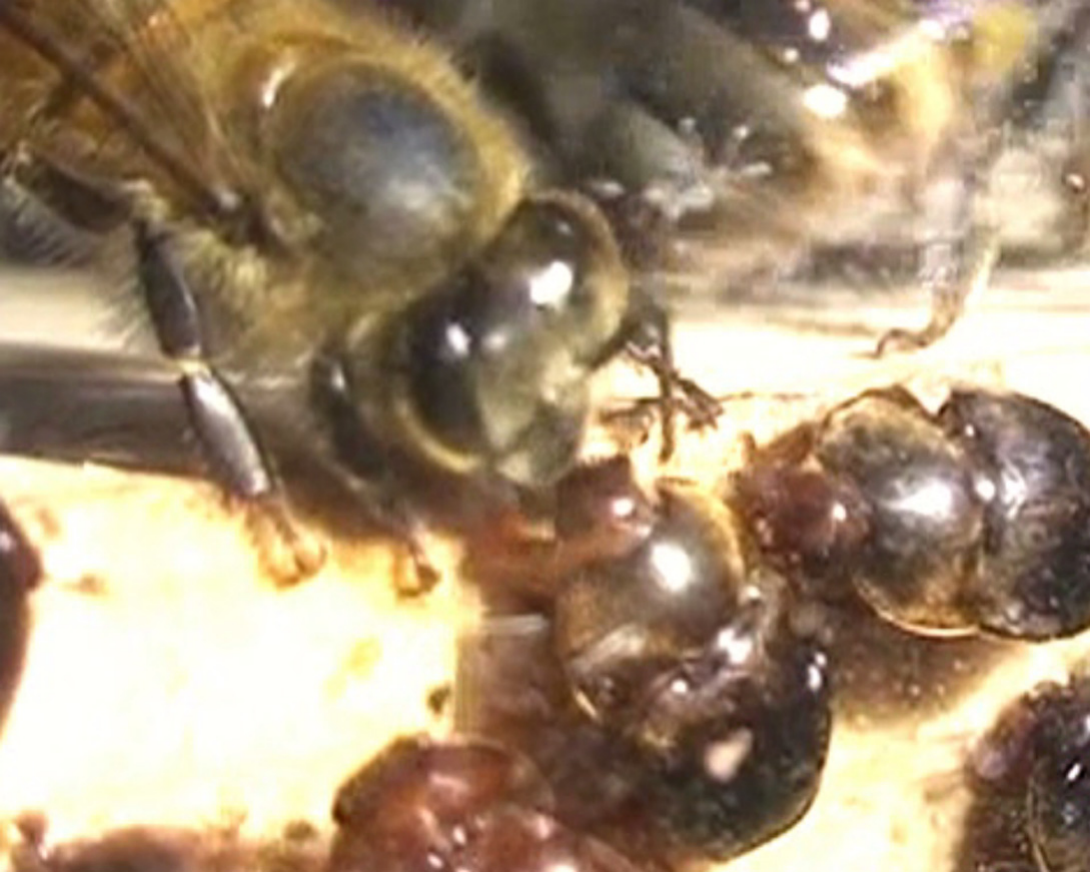
Trophallactic contact between a honey bee worker and a small hive beetle. The screenshot of a movie showing a trophallactic contact between a honey bee worker and an SHB hiding in the gap between Perspex and wood. A second SHB to the right is about to interfere with feeding.

**Figure 3:**
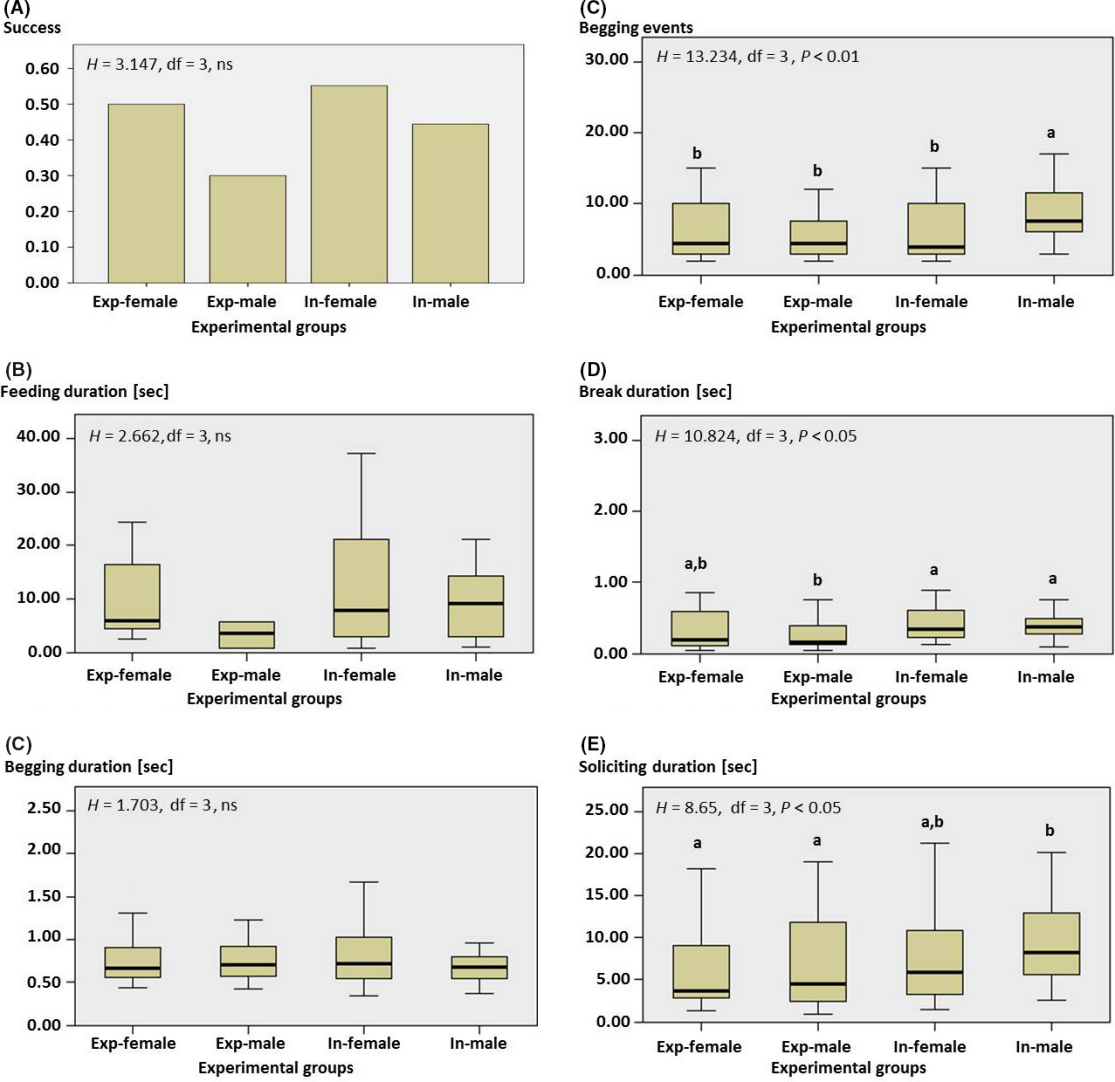
Comparisons of the behavioral interactions between the experimental small hive beetle groups. (A) Success (successful trophallactic events), (B) feeding duration, (C) begging duration, (D) begging events, (E) break duration, (F) soliciting duration. Means (A) or medians, quartiles, and ranges are shown (B–F). Significant differences between groups are indicated with different letters (Exp_female, experienced females; Exp_male, experienced males; In‐female, inexperienced females; In‐male, inexperienced males).

A total of 3823 behaviors were scored and assembled into 110 interactions of which 7 included more than one beetle. The recorded interactions occurred in the 2 mm gap between the Perspex and the wood (see Figs 1, 2). The Kruskal–Wallis ANOVA showed no significant effects of sex and experience on success (*H* = 3.147, DF = 3, ns, Fig. 3A), feeding duration (*H* = 2.662, DF = 3, ns, Fig. 3B), and begging duration (*H* = 1.703, DF = 3, ns, Fig. 3C). However, begging events (*H* = 13.234, DF = 3, *P* < 0.01, Fig. 3D), break durations (*H* = 10.824, DF = 3, *P* < 0.05 Fig. 3E), and soliciting duration (*H* = 8.65, DF = 3, *P* < 0.05, Fig. 3F) differed significantly between the groups (*N* = 29 inexperienced females, *N* = 36 inexperienced males, *N* = 16 experienced females, *N* = 20 experienced males). Inexperienced males begged significantly more often than any of the other groups (*z* > 2.7, *P* < 0.05, Fig. 3). Moreover, break duration of experienced males was significantly lower than inexperienced males, but not for both inexperienced and experienced females (*z* = 2.8, *P* < 0.03 Fig. 3). Lastly, soliciting duration of inexperienced males was significantly longer compared to experienced males and females, but not compared to inexperienced females (*z* = 2.7, *P* < 0.03, Fig. 3). Comparing successful and unsuccessful interactions (Table 1) revealed that soliciting duration (MWU: *U* = 704, *P* < 0.001), break duration (MWU: *U* = 763, *P* < 0.001), and begging events (MWU: *U* = 948.5, *P* < 0.05) were significantly higher in unsuccessful events. Begging duration was not significantly different between successful and unsuccessful interactions (MWU: *U* = 1093, *P* > 0.05).

**Table 1:** Comparison between successful (followed by trophallactic feeding) and unsuccessful interactions between small hive beetles and honey bee workers

	Successful	Unsuccessful	Comparisons
Begging events	5.50 (5.25)	7.00 (7.00)	*
Soliciting duration [sec]	4.29 (5.39)	8.39 (8.73)	***
Begging duration [sec]	0.68 (0.26)	0.68 (0.43)	n.s.
Break duration [sec]	0.24 (0.22)	0.43 (0.42)	***

Medians (interquartile ranges) are shown for begging events, soliciting duration, begging duration, and break duration (*N* = 101). Significant differences between successful and unsuccessful events are indicated with *(*P *< 0.05), ***(*P *< 0.001), or n.s. (*P *> 0.05) using Mann–Whitney U‐tests.

**Figure 4:**
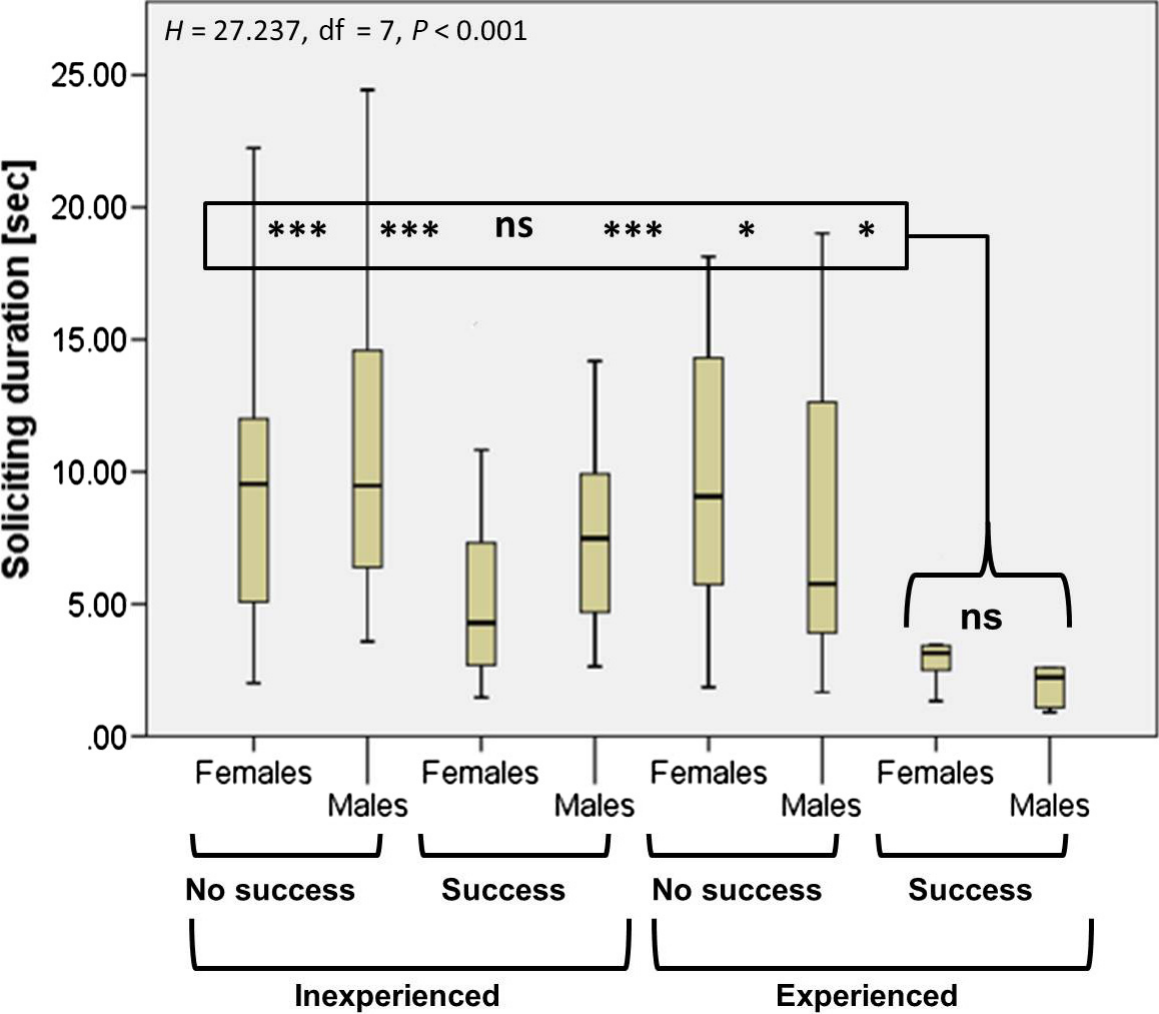
Soliciting duration in the experimental small hive beetle groups. Medians, quartiles, and ranges are shown. Successful experienced females and males were not significantly different from each other, but had a significantly shorter soliciting duration compared to all other groups, except successful inexperienced females. Significant differences are indicated with * = *P* < 0.05, *** = *P* < 0.001, ns = not significant.

Successful experienced females and males were not significantly different from each other in terms of soliciting duration, but had a significantly shorter soliciting duration compared to all other groups, except successful inexperienced females (Fig. 4).

### Interactions with two small hive beetles

In seven of 101 cases, two SHBs solicited food from one bee at the same time. All interactions were successful, and in six cases, both SHBs were fed. Likewise, in six of the seven interactions, beetles were observed pushing each other out of the bee's proximity, a behavior we have termed shoving. A shoving SHB approached its victim head forward from the side and performed 1–3 short forward movements, thereby pushing the other SHB away over a distance of up to 2 cm. Shoving was performed 1–16 times (2.69 ± 3.93) per interaction and was mutual in five of six cases, meaning that both beetles did it.

### Effects of aggression by honey bee workers on small hive beetles

The risk of SHB injury was <1%, because in a single case, a worker was able to bite into the antenna of a begging SHB for 10 sec. Then, the beetle was released, but showed no apparent damage.

## Discussion

The data clearly show that SHB trophallactic solicitation is an innate behavior, which can be influenced by both sex and experience. Overall, success seems to be governed by quality rather than quantity of interactions, thereby probably limiting both SHB energy investment and chance of injury (<1%, 1 case of 108 interactions).

Our results confirm earlier reports about SHB behavioral solicitation (Ellis et al. 2002a) and further show that overall ~50% of interactions are successful. All observed interactions were preceded by a series of begging events separated by breaks, followed by feeding in case of successful interactions. During the breaks, the worker vigorously bit the beetle, confirming earlier studies that SHBs are easily recognized by the host (Elzen et al. 2001). The SHBs evaded these attacks using the turtle‐defense posture (Neumann et al. 2001b) or by retreating from the bees. After the bee's attacks ceased, the beetle usually re‐emerged from its safe position and continued begging. During begging, the SHB moved close to the bee's mandibles, touched them with its own mouth parts anteriorly and proximally. At the same time, it moved its antennae, touching mostly the bee's mandibles, but also its antennae and other parts of the head. SHBs also used their forelegs to touch the bee's mandibles, which is very similar to bee–bee behaviors in trophallaxis (Free 1956; Korst and Velthuis 1982). The observations also showed that SHB and bee mouth parts were in contact prior to feeding. We propose that this not only serves for food uptake, but is an important stimulus in triggering the feeding response, thereby probably being equivalent to the recipient bee's extended proboscis during bee–bee trophallaxis (Free 1956; Korst and Velthuis 1982). Alternatively, but not mutually exclusive, this SHB behavior might also constitute an appeasing behavior similar to that of the myrmecophilous beetle *Pella letticollis* in colonies of the ant *Lasius fuliginosus* (Stoeffler et al. 2011). In sharp contrast to *A. tumida*, the bee louse, *Braula coeca*, is not able to induce trophallaxis in honey bees. Instead, the bee louse takes advantage of two bees feeding each other (Morse and Nowogrodzki 1990). Sitting on the head or abdomen of a worker or the queen, the louse quickly moves forward and steals food during the food exchange between the two bees (Morse and Nowogrodzki 1990). In addition, is has recently been clarified that *B. coeca* uses chemical mimicry to disguise itself in honey bee colonies (Martin and Bayfield 2014). At the current stage of knowledge, it is not clear, whether SHB trophallactic solicitation entirely lacks a chemical basis. For example, the SHB might be able to detect food odors from the mouth parts of the bee prior to “antennating,” because the beetle's ability to detect the alarm pheromone at concentrations undetectable to worker bees has been shown (Torto et al. 2007). It is also likely that similar levels of sensitivity to food odors may be involved here. This might enable an adaptive choice of the SHB, for example, to preferentially target host bees with a low level of alarm pheromone, thereby possibly limiting chances of injury and instead increasing success. Similarly, SHBs seem to be able to discriminate between young and old honey bee workers by assessing the defensiveness of the host and adjusting their behavior accordingly (Pirk and Neumann 2013). However, it is common knowledge that SHBs are readily attacked by honey bee host workers (Elzen et al. 2001), thereby clearly showing that SHBs are at least not as chemically disguised as many other parasites in the social insect colonies are. Nevertheless, the fact that the SHB is readily attacked does not exclude that the beetle has also developed an olfactory mimicking, that is present in parallel. In any case, these issues need to be investigated in more detail in future studies. Therefore, published reports of nonchemically or nonacoustically mediated trophallactic solicitation are currently restricted to a few species, for example, the cricket *M. manni*, which infests western thatching ants (Henderson and Akre 1986) and the SHB, *A. tumida*, when infesting honey bee colonies (Ellis et al. 2002a).

The data clearly show that trophallactic solicitation by SHBs is an innate behavior, because bee‐naïve SHBs were able to successfully initiate feeding by the host workers. This indicates that trophallactic solicitation is important for the SHB survival in host colonies. Indeed, owing to the aggressive behavior of honey bee workers (Elzen et al. 2001), SHB usually hide in cracks and crevices of host colonies, where they are guarded (Ellis et al. 2002a). Such imprisoned beetles may survive for 2 months or longer (maybe also thanks to cannibalism, Neumann et al. 2001b), and their survival is not due to their having metabolic reserves, because starved beetles die within a fortnight (Ellis et al. 2002c). As imprisoned SHBs cannot take advantage of honey and pollen stores as well as brood and hive debris, trophallactic interactions with host bees remain the only source of food, besides perhaps cannibalism (Neumann et al. 2001b), ensuring their long‐term survival in host colonies.

Trophallactic solicitation success, feeding duration, and begging duration were not significantly affected by either sex or experience. This is consistent with previous findings that the propensity of a honey bee worker to feed, as well as the amount of food transferred, depends largely on the donor bee's nutritional state (Free 1956; Crailsheim 1998). The overall high success rate (~50% of interactions) also suggests that SHBs are well adapted to exploit their host's trophallactic interactions and that the donor bees have the right nutritional state.

The longer soliciting and break durations as well as higher numbers of begging events in unsuccessful interactions compared to successful ones, together with no significant differences in begging duration, suggest that successful interactions are distinguished by shorter breaks between the begging bouts and that quality of SHB begging behavior rather than duration of the interaction determine the success of trophallactic solicitation.

Female SHBs have higher protein requirements than male conspecifics, owing to egg production and the often larger body size (Lundie 1940; Ellis et al. 2002c) and consequently have a reduced reproductive output on low protein diets (e.g., fruits, Ellis et al. 2002b; Buchholz et al. 2008). Thus, given SHBs receive jelly from honey bee workers; we would expect females to be superior in trophallactic solicitation compared to males, reflecting the higher benefit they may get from trophallactic feeding, especially when obtaining proteins. Indeed, inexperienced males begged more often than any of the other groups, had longer breaks than their experienced counterparts and a longer soliciting duration than both experienced males and females, thereby suggesting that they start rather slowly and gain more from experience compared to females. The higher success of both experienced and inexperienced females (Fig. 2A) may reflect higher needs of female SHB for food.

Of particular interest were the cases (seven of 101), when two SHBs were soliciting food from one bee at the same time. It appears as if this is not compromising overall trophallactic success, because all interactions were successful and in six cases, both SHBs were fed. Nevertheless, the term “interference” seems appropriate here, because the initiating SHB will have to share with the interfering beetle whatever amount of food will be given by the target bee. Moreover, the observed shoving behavior, when one SHB pushes the other one away using its head over a distance of up to 2 cm, apparently constitutes aggression suggesting that the observed interference is another case of intraspecific competition.

Successful experienced female and male SHBs had significantly shorter soliciting durations compared to all other groups, except successful inexperienced females, suggesting that they have probably achieved a higher benefit to cost ratio by (1) reducing the overall duration and thus the energetic cost of soliciting; and (2) reducing the risk for injury. In a single case, we observed that a worker was able to bite into the antenna of a begging SHB for 10 sec. Then, the beetle was released, but showed no apparent damage. Nevertheless, this observation indicates that the risk of injury is not completely zero and is in line with previously reported rare cases of decapitated adult SHBs (Neumann et al. 2001b). Here, we can actually quantify this risk as being <1% (one case of 108 recorded interactions). In light of the potentially fatal consequences, we still consider this to be a cost for SHB when soliciting food from honey bee hosts. Therefore, experienced SHBs improved key features triggering trophallactic solicitation success, probably via learning. As two SHBs can solicit food from one bee at the same time, exposure cannot be excluded. Through interaction with a conspecific, an animal is exposed to the same learning environment and, therefore, can acquire the same behavior pattern more quickly than it would on its own (Leadbeater and Chittka 2007).

## Conclusions

The data show that trophallactic solicitation of SHBs is an innate behavior and suggest that it can be influenced by both sex and experience. The SHB is a singular example for an alternative strategy to exploit insect societies not requiring chemical disguise and such hit‐and‐run trophallaxis appears to be an attractive model system to better understand trophallaxis in the social insects.

## Conflict of Interest

None declared.
